# Unveiling the invisible: a qualitative interview study on the impact of young onset Parkinson’s disease on (ex-)partners

**DOI:** 10.1007/s00415-024-12474-2

**Published:** 2024-06-11

**Authors:** Willanka Mariëlle Kapelle, Angelika D. Geerlings, Inge Mutsaers, Bastiaan R. Bloem, Marjan J. Meinders, Bart Post

**Affiliations:** 1 Department of Neurology, Donders Institute for Brain, Cognition and Behaviour, Radboud University Medical Center, Center of Expertise for Parkinson and Movement Disorders, P.O. Box 9101 (internal code 914), 6500 HB Nijmegen, The Netherlands; 2 Inge Mutsaers Research and Writing, Nijmegen, The Netherlands

**Keywords:** Young onset Parkinson’s disease, Parkinson’s disease, Caregivers, Quality of life, Qualitative research

## Abstract

**Introduction:**

Living with young onset Parkinson’s disease (YOPD) not only affects the persons with YOPD, but also their families. Although caregiver burden has been researched in Parkinson’s disease in general, little is known about the specific impact of having an (ex-)partner with YOPD. This exploratory study aimed to explore the impact of having an (ex-)partner with YOPD on daily life.

**Materials and methods:**

We used a qualitative approach including semi-structured interviews with 16 (ex-)partners of people with YOPD. Interviews were audio-taped and transcribed verbatim. Qualitative thematic analysis was used to analyze the interview data.

**Results:**

Qualitative analysis revealed nine themes to describe the impact of having an (ex-)partner with YOPD: (1) Psychosocial impact, (2) Impact on taking care of children, (3) Impact on working life, (4) Impact on intimacy and (sexual) relationships, (5) Impact on daily life, (6) Acceptance and other coping strategies, (7) Thoughts about the future, (8) Autonomy and (9) Caregiver support.

**Discussion:**

This study provides important findings that will inform future interventions that aim to reduce or even prevent caregiver burden, as well as to inform healthcare professionals to recognize the needs of caregivers of people with YOPD.

**Supplementary Information:**

The online version contains supplementary material available at 10.1007/s00415-024-12474-2.

## Introduction

Parkinson’s disease (PD) is a chronic and progressive neurodegenerative disorder characterized by both motor and non-motor symptoms [1]. Although the prevalence of PD rises sharply with age, with a mean age at diagnosis between 70 and 75 years, about 5–10% of all people with PD experience their first symptoms under the age of 50. This is referred to as Young Onset Parkinson’s Disease (YOPD) [2]. PD has a major impact on those living with the disease, as well as on those caring for the person with PD. As the disease progresses, people with PD become more dependent on their social environment and require more assistance and support with activities of daily living from their informal caregivers, who are typically direct family members such as partners or children [3]. For people with YOPD, the role of informal caregiver is often taken on by a young partner.

Informal caregiving has generally been associated with burdensome experiences given the physical, mental, relational and economic stressors of providing care for a person living with PD [4–6]. Informal caregiving has been linked to poorer reported health outcomes, psychological stress, depressive symptoms and a poorer quality of life [5, 7–10]. Previous research in oncology revealed that age plays an important role in perceived caregiver strain, since young, middle-aged and older informal caregivers experience different priorities in life [11]. Becoming an informal caregiver at a younger age can interfere with the demands that this life phase puts on younger people, such as completing education, entering the workforce, striving for career, establishing intimate partnerships and raising children [12, 13].

Few studies have assessed the impact of providing informal care to a person with YOPD. Most studies focused on middle-aged or older caregivers [14, 15], which does not capture the specific challenges that come with being a younger caregiver. In addition, previous YOPD-specific studies have only focused on quantitative methods to examine the impact of caregiving on perceived burden in people with YOPD [9, 13, 16]. To examine the unique experiences of younger informal caregivers providing care for a person with YOPD, this study used a qualitative approach by interviewing partners as well as ex-partners of people living with YOPD. A better understanding of the impact of having an (ex-)partner with YOPD will help to develop tailored interventions to improve care and support for younger (ex-)partners of people with YOPD.

## Materials and methods

### Design and participants

This study was part of a broader study exploring the impact of YOPD on the daily life of people with YOPD, their (ex-)partners and children in the Netherlands through the use of qualitative, in-depth, semi-structured interviews. Within this broader study, we first enrolled 50 people with YOPD. They were recruited through the outpatient clinic of the neurology department of the Radboud University Medical Center, through an advertisement on the website of the Dutch Parkinson Patient Association, among participants of an earlier clinical study who had consented to be approached for other research and through the personal networks of people with YOPD who were involved in this project. All people with YOPD were diagnosed with idiopathic Parkinson’s disease between the age 21 and 50 years, confirmed by a neurologist. Suitable participants were selected in an iterative manner, based on characteristics that ensured a maximum variation in the study sample in terms of employment status, gender, age, disease duration since diagnosis and level of education.

For the purpose of this particular study, we focused on (ex-)partners of people with YOPD. This study was designed as an exploratory qualitative study using in-depth interview with current partners as well as ex-partners of people with YOPD. We included ex-partners of people with YOPD to broaden our understanding of the impact PD can have on the relationship. We reached out to (ex-) partners through the people with YOPD who had previously participated in this study, as well as through social media, healthcare professionals from other medical centers, personal networks of people with YOPD (e.g., sports clubs and Parkinson’s cafes), databases of other studies and key players in projects that specifically target diversity and intercultural aspects within Parkinson’s research. In addition, we have reached out directly to members of the Islamic community in the Netherlands. A letter with information about the study was sent to potential participants via email, in which they were invited to participate in a one-time interview regarding the impact of YOPD on their life. (Ex-)partners of people who received their PD diagnosis by a neurologist between the ages of 21 and 50, who were able to speak and understand Dutch and where 18 years or older were eligible to participate. Participants were selected iteratively, to ensure variation between the participants in terms of gender, disease duration of their (ex-)partner, relationship status (ex-partner vs current partner) and level of education. In total, we invited 36 people with YOPD to include their (ex-)partners in this study of whom sixteen could be included; eight (ex-)partners did not respond to our invitation; nine (ex-)partners expressed having no interest in participating due to traveling distance, lack of time, seeing no relevance in participating or because of a recent ending of the relationship with the person with YOPD; two partners did not speak Dutch; and one partner could not participate due to own illness. Among the (ex-)partners who were not included were seventeen current partners (57% of all invited current partners) and three ex-partners (50% of all invited ex-partners). No relationship was established between the researchers and participants prior to the study commencement and participants had no prior knowledge of the interviewer.

### Data collection

An exploratory, qualitative study using semi-structured, face-to-face interviews among sixteen (ex-) partners was undertaken. Data collection took place between July 2021 and January 2022. An interview guide was developed based on a literature review [2, 6, 13, 16–22] and experiences from clinical practice of one of the authors [BP, MD (neurologist), PhD, male]. The interview guide was piloted in the first two interviews. Later on, if new topics emerged from interviews, these were added to the guide for the remaining interviews. The final version of the interview guide is included in Online Resource 1. The guide consisted of three parts covering different life stages: (1) diagnosis, (2) current situation and (3) future expectations. The interviews were conducted by an independent researcher [IM, female], biologist (MSc), trained in journalism, with a PhD in Philosophy and Science Studies. Each interview lasted between 67 and 143 min and was conducted in the participants' native language (Dutch). The interviews took place at the Center of Expertise for Parkinson and Movement Disorders at the department of neurology, Radboud university medical center Nijmegen, the Netherlands. This way, interviews could take place without interruptions, in a secure place and the interviews could be recorded using professional audio-visual equipment, which resulted in high quality recordings. Moreover, this setting guaranteed that participants were able to speak freely about their emotional well-being and current, as well as past experiences without the partner being present. Interviews were both video- and audio-taped for quality purposes and field notes were taken during the interviews. Transcripts were not returned to the participants prior to data analysis.

### Analysis

All audiotapes were transcribed verbatim and entered into ATLAS.ti version 9.1.6 for content analysis following the principles of thematic analysis. The transcripts were coded and analyzed simultaneously by two authors [WMK, female, researcher, MD; ADG, female, researcher]. We used thematic analysis to analyze our data [23]. Data analyses were conducted concurrently with data collection. During consensus meetings the codes were compared and discrepancies or disagreements during the data analysis process were solved. Initially, open coding was used, followed by axial coding to compare relationships between the used codes, from which broader themes emerged. Emerging themes were continuously discussed with the co-authors. Data saturation was reached after thirteen interviews, when coding and analyzing additional interviews did not lead to any new insights. Three more interviews were conducted to ensure data saturation. Participants did not provide feedback on these findings.

### Ethics

The study was performed following the ethical guidelines of the Declaration of Helsinki, version 2013. The ethical board of the Radboud university medical center (file number 2023-16174) reviewed the study protocol and confirmed that no further ethical approval needed to be obtained. All participants provided written informed consent.

## Results

### Participant characteristics

The study was conducted among thirteen partners and three ex-partners of people with PD. The participants' characteristics are summarized in Table 1. Half of the (ex-)partners (9/16) were female, with caregiver ages ranging between 33 and 67 years. Disease durationvaried between 0 and 17 years. In the result section, the (ex-)partners of people with YOPD are referred to as ‘participants’; the people with YOPD are referred to as ‘(ex-)partners’.

**Table 1:** Characteristics of the participants and their (ex-)partners with YOPD (*n* = 16)

	Characteristics participants	*N* (%)	Mean (SD); range	Characteristics (ex-) partners with YOPD	*N* (%)	Mean (SD); range
Gender	Women	9 (56.3)		Women	7 (43.8)	
Cultural background	Western European	15 (93.8)		Western European	15 (93.8)	
	Eastern European	1 (6.3)		Eastern European	1 (6.3)	
Relationship	Married	10 (62.5)				
	Divorced	3 (18.8)				
	In a relationship	3 (18.8)				
Relationship at diagnosis	Yes	14 (87.5)				
Children	Yes	11 (68.8)				
	No	1 (6.3)				
	Blended family*	4 (25.0)				
Children younger than 12	Yes	2 (12.5)				
Children younger than 18	Yes	10 (62.5)				
Children living at home	Yes	12 (75.0)				
Number of children						
	0	1 (6.3)				
	1	4 (25.0)				
	2	4 (25.0)				
	3	5 (31.3)				
	< 3	2 (12.5)				
Paid job	Yes	13 (81.3)		Yes	9 (56.3)	
Education level**	Low/Middle	2 (12.5)		Low/Middle	3 (17.8)	
	High	14 (87.5)		High	13 (81.3)	
Age (years)						
	31–40	3 (18.8)	50 (± 8.0); 33–67	31–40	2 (12.5)	49.8 (± 6.9); 34–64)
	41–50	6 (37.5)		41–50	6 (37.5)	
	51–60	6 (37.5)		51–60	7 (43.8)	
	61–70	1 (6.3)		61–70	1 (6.3)	
Age at diagnosis				31–40	4 (25.0)	42 (± 4.9); 31–48
				41–50	12 (75.0)	
Hoehn & Yahr stage				II	13 (81.3)	
				III	1 (6.3)	
				IV	1 (6.3)	
				Unknown	1 (6.3)	
Use of PD medication				Yes	15 (93.8)	
Deep brain stimulation				Yes	4 (25.0)	
Duodopa or apomorphine pump				Yes	2 (12.5)	

*Have children separately, but not together **Based on the Dutch educational system; low: primary education or low-level professional education; middle: secondary education or medium-level professional education; and high: tertiary education (bachelor degree or higher)

### Qualitative themes

**Fig. 1:**
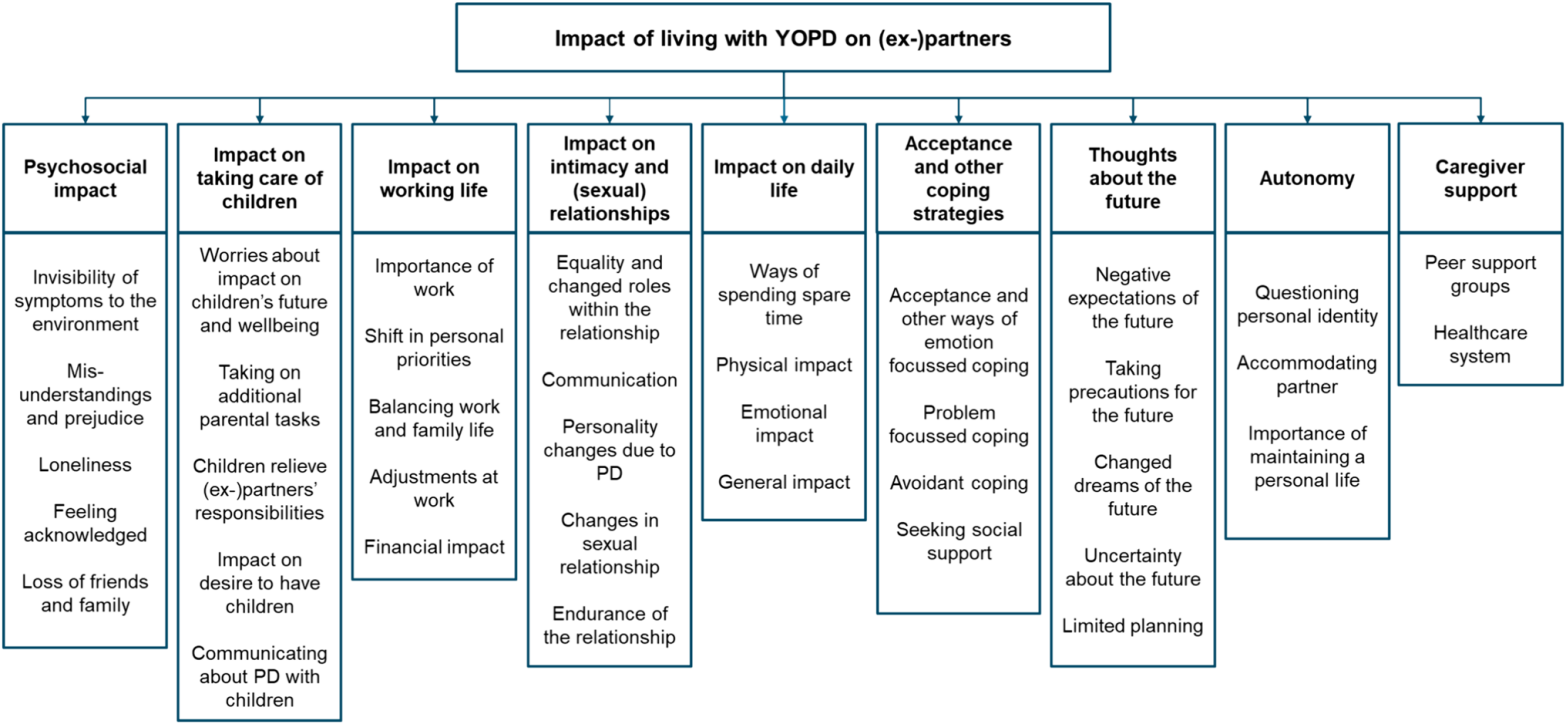
Summary of key themes

Nine key themes were identified: (1) Psychosocial impact, (2) Impact on taking care of children, (3) Impact on working life, (4) Impact on intimacy and (sexual) relationships, (5) Impact on daily life, (6) Acceptance and other coping strategies, (7) Thoughts about the future, (8) Autonomy and (9) Caregiver support. The key themes and sub-themes are summarized in Fig. 1 and illustrative quotes are given in Table 2.

**Table 2:** Illustrative quotes*

Key theme	Illustrative quotes	
1	Psychosocial impact	*“I noticed that a lot of people find it difficult to deal with the situation. Whether they should ask some questions or not. I also talked about it with my partner […] and said that you should tell people how you feel […] what you find difficult. Then people can develop understanding. […] And I also try to tell people that they see him at good moments […]. You can see a broken leg. The fact that someone moves in a different way. You can see it. But you do not see the inner changes.” [P12]*
2	Impact on taking care of children	*“I have always just tried to really be there for [my daughter], because it was harder with [my ex-partner]. So maybe you just become a little more worried over your daughter and that is why – Yeah, maybe you just want to be there for your daughter, so to say.” [P07]*
		*“If, at first, we only had a few good years to secure our children. After that, we will see. [Our son] is 10 now. Well, for the next 10 years I feel like we are going to be fine. But after that, I am not sure anymore.” [P15]*
		*“Well, you need to go on with it, especially when you have young kids. Then it is really difficult and heavy. My kids are older now. My eldest son is 17 and I have a 15-year-old daughter […]. I do try to get them to lend me a hand […], especially when I am no able to be present […] for a few days. Because it happens that I am skiing with colleagues or a friend for some day’s […]. Now it is easier. My children were growing up with it. So, they know what is going on […] They do not have to help all the time, but it is very nice to see that they do it automatically […]. And then you come home from four days of winter sports with my colleagues, and I hear that my children did a fantastic job […] and helped and made breakfast […] Years ago that was a lot more difficult. Heavier.” [P10]*
3	Impact on working life	*“I used to be the CEO of a large company. I had to be there all the time. [My ex-partner] used to come with me, to concerts and parties and things like that. At a certain moment, after that, I have started working at a smaller company, so that I could have a little more influence over my own agenda in order to have a little more freedom. So that is what I do now. […] If you are the boss of a large company, eighty percent of your agenda is determined by other factors. Then you cannot just say ‘I have to go home’ or something like that if you need to. So, I think that played a role in me aspiring less.” [P07]*
		*“I am not that ambitious anymore” [P12]*
		*“Of course, I am lucky that I can work from home a lot […] But sometimes I struggle with the forty hours [of work]. Is it rational to work forty hours? Especially, when you find yourself in a phase where you want to spend time together to do things together”[P10]*
4	Impact on intimacy and (sexual) relationship	*“If he is getting worse […], then I do not see him as a partner anymore […]. Then I can no longer see through his Parkinson’s. Then I am observing.” [P02]*
		*The relationship became less equal” [P07]*
*“*		*“He used to be more enthusiastic, showed much more initiative. He came up with ideas by himself. He once brought a present or flowers or even arranged a dinner. That does not happen anymore” [P04]*
5	Impact on daily life	*“I have always made sure I stayed physically healthy. Kept exercising, kept doing things. I do think that it all has an impact on your mental state […] You are always very involved with your partner with what you are doing and how things are going at home. But […] the world becomes smaller.” [P07]*
		*“I had many conversations with my children about what [behavior] stems from their father and what from his Parkinson. And how do you deal with that” [P10]*
6	Acceptance and other coping strategies	*“Parkinson’s disease is a disease of loss” [P12]*
		*“Do not put too much emotion into it. Just try to talk like it is the most normal thing in the world. Even though it is often very difficult, but it is that acceptance that you must find” [P10]*
		*“You always have to build up resources, because it can always happen that my partner cannot or will not make it […] On the one hand you really feel small, but on the other hand you feel like you have developed those big shoulders” [P12]*
7	Thoughts about the future	*“Especially in the business world where it is important to make a career. Working towards your future. Where do you want to be in 5 years? […] Now you are very much forced to think about what you want to do with your career […] But maybe in five years I will spend three days per week at home in the role of a caregiver” [P10]*
		*“When will I stop working? When will my partner stop working? […] There are also sad moments when you think 'Why do I have to think about this?'. You just want it to be different […] Like my parents. I want to retire at the age of 68 or 70 and then I want to enjoy things and travel and maybe buy a camper. […] But at a certain point you think differently. We need to have money for a care home or for home adjustments. Those are pretty hard times.” [P10]*
8	Autonomy	*“I do feel [affected in my autonomy]. In my own autonomy as well. I have adjusted. Like I said before, in my job. It is not that I am a very career-oriented person, but in doing things. I used to do street theat. Now I have a hard time standing there, having fun. Then I feel like, hell, I cannot do this anymore.“ [P09]*
		*“I would be willing to defend that as a general principal for informal care takers, that it is very important to have the time to do the things that you want to do. And that is not selfish or anything, but rather altruistic, in the sense that you will be better at carrying out the task of supporting someone. [ …] I would say that I am selfish enough to keep on doing the things that I like, that are important to me.” [P01]*
		*“Sometimes you are forced to make adjustments. We had to do it quite a few times. And that is sometimes very painful, a bereavement. […] I am feeling sad about the thought that my partner cannot make that experience anymore” [P02]*
9	Caregiver support	*“I think in an ideal situation, you should look as closely as possible at the family, like, can everything carry on the way that it did? […] It would be a good question to ask at the start of the trajectory. […] It is a very good question to ask. And to figure out how you can carry on as a family. Or carry on, how you can keep on functioning in this kind of situation.“ [P16]*
		*“What I long for, my biggest fear is that I am inclined to carry on for too long. With [my partner’s] work I think that, retrospectively, we carried on for too long, pushed for too long. He did and I did as well. […] And indirectly, my daughter probably did too. That someone monitors the family independently, in all stages of [the trajectory]. Like, ‘I see you’. But who also monitors the caregiver. And also monitors the child, if they are still living at home. But also at the start, like ‘You have just received a very difficult message. I would handle it like this’. […] And who asks you some questions. ‘Are there any needs?’ ‘Do you know what is going to happen?’. Or when you are sad, ‘Are you going to get through this together?’. That kind of questions.” [P09]*
		*“And then I’m getting back to this independent party that I am hoping for so much. That is my only wish for the entire Parkinson's trajectory, who both looks at ‘What is right for the patient?’. And if necessary ‘What is right for the entire family?’ […] And ‘What is right for the partner?’, who may or may not be present. […] I mean, in our situation, what is right? […] What can you handle? ‘I can see you are suffering. How do you organize that?’ Or ‘Hey, you are still working. How do you find your balance?’” [P09]*
		*“And that’s why I come back to my coach ofr personal trainer or whatever you’d like to call it, but someone who screens the entire family. You know, for a day. Let’s see how that goes. What is happening to the children? You know, that kind of things for example – At some point, there was a shift in [her] sleep pattern. So, it would happen that at 9 AM she would still be asleep, and the kids would already be at school. Well, they are fine preparing their own sandwiches. But there are other families where that may not be the case, because they have young children. But what does a day look like? […] How is mom? How is dad? What are they doing? You know, those kids know even more than I do. You know, they will tell the truth right away. That’s another advantage. So, I think there is still a lot to gain there.” [P03]*
		*“We went to this [peer support] meeting and we thought it was a very heavy-hearted meeting. They showed all kinds of scientific research, that shows/showed that we are very far away from a solution. So, you can go there, but that is not how I want to live.” [P16]*
		*“I think that [during the peer-to-peer contact] really good conversations take place […] It is about the depth. Recognition […] and it is also great to be able give advice to someone else.” [P10]*

* Quotes have been translated from Dutch to Englis

### Psychosocial impact

In describing the impact of PD, participants referred to PD as sometimes being an ‘invisible’ or ‘hidden’ disease, especially the presence of non-motor symptoms, including feelings of anxiety, depressive symptoms and fatigue. Those symptoms are not immediately visible to others and make it more difficult for others to understand the impact of these symptoms on the person with PD, but also on the participant. Participants reported that sometimes, the impact that PD had on their daily lives, e.g., having to take on caregiving tasks, the shift in role division and changes in the relationship, was invisible to others. Participants also associated this lack of visibility of PD-related symptoms with a lack of understanding from the environment. This manifested itself by prejudices or misinterpretation and the felt need of participants to explain the impact of PD to others. Participants also expressed that friends and relatives often found it difficult to have a conversation about PD. Even if friends or relatives inquired about the impact of their (ex-)partner’s PD on their lives, they usually just inquired about the person with PD and not about the participant. This often led to participants feeling lonely and unacknowledged. In some cases, the (in-)visibility of PD to the environment could change relationships with family and friends or even cause a loss of friendships.

### Impact on taking care of children

Participants recurrently talked about the impact of PD on raising their children. Although PD was generally not a daily topic of conversation or even a topic that was kept away from children, participants expressed worries about the impact that their (ex-)partner’s diagnosis might have on their children’s future development. They wished for their children to be able to live their own lives and often expressed relief once their children had grown up to be adults. Some participants reported consulting with a therapist to ensure their children’s psychological well-being. Participants generally took on a larger role in taking care of their children, to compensate for the care in which their (ex-)partners were not able to fully provide. Participants who were former partners sometimes experienced an added burden, since they could feel like they still had to take care of their ex-partner to ensure their children’s well-being. This was especially the case when the children were of younger age. Participants reported that children at an older age could also take over some care tasks and thereby provide (ex-)partners with time for themselves without feeling guilty that they had left their partner alone. Moreover, our data revealed that a PD diagnosis could play an important role in the decision-making process for couples who still had a desire to have children. Participants reported that along with the wish for (more) children, they also had some doubts about the possibility of the (ex-)partner to take care of the child, especially when motor symptoms became more severe. They reported that they were aware that taking care of the child(ren) might become their responsibility and even small tasks, such as changing diapers, would become difficult for the partner due to motor impairments.

### Impact on working life

Having an (ex-)partner with YOPD often resulted in changes at work. Although work was important for stability and a secure financial situation, participants often mentioned that having an (ex-)partner with PD had caused a shift in their personal priorities. The notion that PD symptoms will progress over the years led to the decision to spend more quality time with their (ex-)partner and family over pursuing a career. Moreover, taking care of an (ex-)partner with PD made it harder to balance both work and family life. This often led to participants eventually opting for a less demanding job, a reduction in working hours, a job closer to their home or working from home more often. Although PD sometimes influenced participants’ financial situations, e.g., because their partner had lost their job, all participants reported that they were currently in a secure financial position.

### Impact on intimacy and (sexual) relationships

One recurring theme that emerged from the analysis related to changes that occurred in the relationship of the person with PD. This included the experienced struggle to find a balance between being a partner and being a—sometimes in an unwanted way—caregiver to a person with PD. As the disease progressed, participants reported experiencing a role change as they were forced to carry out more caring tasks and take over responsibilities, including household and financial duties. Moreover, participants reported sometimes taking over too many duties out of worry for their (ex-)partner’s wellbeing, which could unnecessarily accelerate this role change. In addition, participants reflected on the way their role had changed from being an (ex-)partner to someone with YOPD to being an “observer” of their (ex-)partner’s symptoms, partly boosted by the fear of worsening motor and non-motor symptoms. Some participants stated that PD became ever-present, and the partner had moved towards the role of the “patient”, especially with considerable worsening in cognitive functioning and severe motor impairments. Open and honest communication was reported to be essential to maintain closeness and connection within the relationship, but PD symptoms could also contribute to difficulties in communication. Participants expressed their sadness about the loss of an equal relationship and how this had affected their intimate relationship with their partner. Moreover, participants reported personality changes in their (ex-)partners, such as loss of initiative, loss of empathy, anxiety, mood disturbances and impulse control issues. These personality changes could be the result of either PD or a side effect of medication. The partner was not the partner they were used to, which could cause tension in the relationship and could even contribute to the relationships ending.

Participants mentioned changes in their sexual relationship with their (ex-)partner, which could be accompanied by reduced closeness to their (ex-)partner or even feeling like they had lost their (ex-)partner. Some symptoms, such as motor and speech impairments, as well as increased dependence and insecurities, could cause participants to feel less attracted to their partner. On the contrary, some participants reported that PD had helped them to develop more closeness to their partner with PD as they spent more time with each other, undertaking activities together and with increased mutual understanding.

While some participants reported that they had never had doubts about staying with their partner or had even chosen to start a relationship with their partner after they had already been diagnosed, others expressed that they had wondered whether the relationship would hold throughout the disease trajectory. These participants were worried that they would not be able to keep up with the added burden of the disease, but also reported a stigma attached to considering leaving a partner who has been diagnosed with an illness. Some relationships eventually ended. An important reason for relationships ending was a shift in what used to be shared priorities, needs and wishes. Changes in the relationship due to PD aggravated issues that had often already been present beforehand.

### Impact on daily life

Participants reported three ways in which their (ex-)partner’s diagnosis impacted their daily life: (1) ways of spending spare time; (2) physical impact; (3) emotional impact.

#### Ways of spending spare time

Loss of energy, loss of flexibility and need for structure are important features of PD and participants reported adapting to their (ex-)partner’s limitations and preferences to still undertake activities together. As a result, participants became more selective in planning how to spend their spare time, to spend as much quality time with their partner as possible. They also reported undertaking more activities alone. This loss of shared experiences could result in participants experiencing a loss of closeness to their partner. Moreover, participants often mentioned that relationships with friends and family changed, because they would not be able to undertake as many activities together as before.

#### Physical impact

Participants stressed the importance of personal fitness and health to be able to fulfill their role as an informal caregiver. They reported that they often took on more roles and tasks, therefore, putting a larger strain on their body, which could result in physical discomfort. Participants expressed maintaining a healthier lifestyle to be better equipped to take on this extra strain, e.g., by becoming sportier and maintaining a healthier diet.

#### Emotional impact

Participants mentioned the emotional impact of their (ex-)partner’s diagnosis. Participants often went through a grieving process after impactful moments, such as the diagnosis or noticeable changes in the relationship. Sadness and anger were mentioned as predominant emotions. PD was ever-present and a constant factor for worry in their lives, impacting participant’s sleeping quality and overall physical health. Contrarily, participants also reported being more sensitive to other people and therefore emotionally connecting to people more easily.

Even though participants noticed the impact of YOPD on their ways of spending spare time, as well as the physical and emotional impact, many reported that, in general, they did not experience this impact to be a large burden, due to the graduality of changing circumstances.

### Acceptance and other coping strategies

Participants had developed various coping strategies to deal with PD. Acceptance of PD and its impact was a recurrent theme in the interviews. It was linked to a process of letting go of the past and learning to accept the changes PD had brought into their lives, including loss of freedom and loss of initiative from the person with PD. Moreover, the process of accepting PD was described as a process of accepting getting a new identity as a caregiver, which was also part of dealing with PD as a couple. Participants reported having assessed what is still possible while living with a person with YOPD and what they had to let go. For instance, they had considered to stop working or to move to a new house that, e.g., would be accessible for a person with more severe motor symptoms, or had a smaller garden. In this process, many expressed a strategy of focusing on possibilities instead of limitations, although they recognized the risk of downplaying the impact of PD. Some reported actively looking for extensive, reliable information on PD as a coping strategy. Others reported that they found comfort in social interactions with others who had had similar experiences and had a common understanding of what it is like to live with a person with PD. In some cases, seeking help from others was accompanied by engagement in activities, such as walking or yoga that helped to clear their mind. Apart from seeking social support from peers, friends or family, participants also shared their thoughts, problems and feelings with others who were not that close to them, such as colleagues, or chose to organize their thoughts by writing them down. While some participants mentioned that they had been avoidant about facing the impact of YOPD on their daily lives, most agreed that they did not consider this a sustainable strategy to cope with their situation.

### Thoughts about the future

Informal caregivers reported concerns about the unpredictability of the future, on the lives of their partners living with YOPD as well as on themselves. Worsening of motor and non-motor symptoms and facing their own personal limitations of what they were capable to uphold in the role as an informal caregiver were the main expressed concerns. Many participants reported being most concerned about their partner developing non-motor symptoms, such as dementia. They often wondered for how long they would be able and willing to prevent their partner from being admitted to a nursing home when symptoms would get more severe. As PD symptoms continuously changed and got more severe over time, participants reported continuously needing to adjust their life to adapt to the current situation. Sometimes, these adjustments meant that they had to give up on dreams for the future, such as buying a bigger home or travelling after retirement. Some participants talked about loss of control and inability to plan when thinking about the future. At the same time, participants also reported that they regained some sort of control by making some adjustments regarding their living situation, e.g., by selling the house and living in a flat that is more suited for someone with motor impairments, by changing their job so that combining work and providing care in the future would be easier, or even by already discussing their partner’s end of life decisions. Other participants reported their strategy was to live a “normal” life as far as possible by maintaining work and home life and planning vacations and social activities together. These participants intended to live in the moment as much as possible and not focus on their uncertain future. They stressed the importance of not postponing any activities that they still wanted to experience as long as they had the possibility and to enjoy the quality time that they could still spend together. This allowed participants to feel like PD did not fully take control over their lives.

### Autonomy

Participants experienced a loss of personal autonomy, with some participants wondering how to balance their role as an informal caregiver with finding time for personal development and social activities. This was caused by continuous worry about their (ex-)partner’s wellbeing and feeling the responsibility to continuously be accommodating to changes in their partner’s health. Although difficult, participants emphasized the importance of continuing to exercise hobbies and spend time on personal development, since they felt like this eventually made it easier to maintain their role as a caregiver.

### Caregiver support

#### Peer support groups

Participants had varying experiences regarding peer-to-peer contact with other informal caregivers. Peer-to-peer contact, as a coping strategy, helped some participants to process their own experiences and find a safe place to share their thoughts and emotions with others. Moreover, participants found peer-to-peer contact to be a source of information about the disease, how to deal with signs and symptoms and to find additional support resources. However, for peer-to-peer contact to be helpful, participants had to be able to empathize with their peers. Important barriers in empathizing with peers that were reported among participants were differences in coping strategies and not being in the same age range. Several participants did not have any contact with peers, for two reasons: (1) Not succeeding in finding any peers in their proximity, and (2) a lack of desire to meet with peers. Lack of desire to meet with peers was often caused by the pre-assumption that they would not be able to reach any peers in the same age range or that they would not personally gain anything from peer-to-peer contact. In addition, some participants reported that they did not want to be confronted with what might become their reality in the future. Those participants reported that they did not want to hear from peers how severe motor and non-motor symptoms could become and impact their life. Others reported that, although they did not feel the desire to reach out to peers in the early stages of the disease, this changed once the impact of PD on daily life became more prominent.

#### Healthcare system

Regarding formal care, participants stressed the importance of taking the impact of the disease on the (ex-)partner into account. Healthcare professionals should pay attention to the (ex-)partner’s well-being, especially during impactful events in the disease trajectory, e.g., diagnosis or deep brain stimulation. Receiving adequate information about the impact of YOPD, treatment options and disease progression was essential to participants, allowing them to carry out their tasks as an informal caregiver, as well as seek help for themselves if needed. For this information to be beneficial, it should be tailored to the (ex-)partner’s specific situation. Moreover, participants expressed the desire for a personal professional that would monitor the daily impact of YOPD on the lives of both the person with YOPD and their families, to provide personalized advice on how to reduce this impact. With the neurologist and Parkinson nurse often mainly focusing on medication and physical symptoms, attention to the psychological impact of the disease on the family in general and the partner, in particular, was identified by many participants as an important gap in the healthcare system.

## Discussion

Our study builds on previous studies exploring age-related differences in experiencing the impact of living with a person with a chronic disease [12, 13]. While there have been previous studies exploring the caregiver burden, perceived quality of life and coping strategies of informal caregivers of people with PD in general [4–6], this study focused on the lived experiences of current and former partners of a specific and hitherto poorly studied subgroup of people with PD.

### Multifaceted PD: Unveiling the invisible visible

One of our main study findings was the expressed desire and need of current and former partners of people with YOPD to receive a better understanding of their situation from their environment, including family, friends and colleagues. PD is often associated with people of older age, which can cause stigmatization and misunderstanding of people being diagnosed with PD at a younger age. In addition, not all symptoms and signs of PD are visible on the outside, and people with PD are mostly seen when medications are working well, and when the symptoms are relatively well under control. The environment might therefore easily underestimate the impact of YOPD on the partner. To correct this, it is important to change the image of PD in popular media, scientific research and clinical practice. Depicting a more diverse and inclusive image of people with PD and their symptoms could change the public perception of people with YOPD and their caregivers [24].

### Age differences in experiencing impact of PD

Receiving a diagnosis at a younger age comes with different challenges to the person with PD as well as the partner, as compared to those receiving the diagnosis later in life. The diagnosis is made during a phase of their lives during which people usually develop themselves, e.g., by establishing their career, reaching personal goals, and starting a family. Not surprisingly, research revealed that people with YOPD are at higher risk of non-motor symptoms, including anxiety and depression [25], and psychosocial problems, including breakdown of relationships and inability to continue to work [26]. Our results show that the impact of living with PD does not only differ with age when it comes to people with PD but also to their (ex-)partners. Previous research on the impact of PD on those living with the person with PD revealed loss of freedom, feelings of isolation and lower perceived quality of life [14, 19, 27, 28].

Comparison of our study findings with research published on people with PD who were diagnosed later in life indicates similarities as well as differences. Consistent with findings reported in a previous study with informal caregivers of people with PD, our findings revealed the struggle of feeling misunderstood caused by the invisibility of PD [14], experienced loss of autonomy [15], and concerns about the future impact of PD on the partner’s life [14]. However, our results also revealed the different impact of PD on relationships and employment of (ex-)partners of people with YOPD. Regarding the relationship, (ex-)partners often struggled with finding a balance between being a caregiver and being an (ex-)partner, dealing with personality changes in the person with YOPD and in accepting the loss of closeness to the person with YOPD. Regarding working life, they expressed that living with a person with YOPD had shifted their priorities from work to family life, with many (ex-)partners adjusting their current jobs and future career paths. Another important age-related challenge was the added burden of taking on extra responsibilities regarding raising children.

### Need for psychosocial support

In addition to education and information needs about the impact of YOPD, treatment options and disease progression, our study revealed that (ex-)partners are searching for psychosocial support in dealing with the disease. There is a clear psychological component to each of our nine identified themes. Based on our analysis, we suggest the following options for providing psychosocial support: (1) Counseling about for instance work- or family-related problems, (2) Peer support groups, and (3) Having access to a dedicated personal professional.

#### Counseling

Although receiving psychological counseling is rather common for people with YOPD at some point in the disease trajectory, participants in our study indicated that they often did not receive any kind of counseling. It is important to involve (ex-)partners in this counseling process as well, to help them cope with challenges they experience in the relationship, grief, experienced loss of autonomy and worries about the future. By offering partners counseling at an early stage in the disease, they could be better prepared for what to expect when the disease progresses [29]. Pinpointing potential pitfalls for informal caregivers of people with YOPD could prevent problems in the future [30].

#### Peer-to-peer support

Peer-to-peer support can play an important role in coping with the impact of having an (ex)-partner with YOPD by sharing aspects of the impact of PD and easing feelings of loneliness. As previous research revealed, peer-to-peer support can offer caregivers of people with PD a safe place to share emotions and thoughts, to receive recognition and understanding, and to receive information and practical advice [31, 32]. A recent study also revealed the potential of using former informal caregivers of whom the partner with PD deceased to take on the role as an expert to support current informal caregivers [33].

Although peer support can be of great value to caregivers of people with YOPD, it should be adjusted to their specific needs and wishes. To benefit from peer support groups, participants should be in the same age range and YOPD-specific difficulties should be discussed. Moreover, peer support should be organized in different ways, e.g., through real-life one-on-one meetings, real-life group meetings or online support groups. Providing various possibilities for peer support could lower the bar for (ex-)partners of people with YOPD to reach out to peers.

#### Having access to a dedicated personal professional

Previous research showed the need for people with PD to be able to have ready access to a healthcare professional as a single point of contact who could act as a personal case manager to address the problems of people with PD, either directly or by referring them to other health care professionals [34]. Our results show the additional need for (ex-) partners of people with YOPD for a personal professional to monitor the impact of YOPD on the family as a whole. Being a caretaker made it difficult to perceive and take care of both their own needs and the needs of the person with PD and their children. Caregivers of people with YOPD could potentially benefit from a personal professional to monitor their personal, at-home situation to provide them with personalized advice on overcoming the barriers they face. We feel that research into the merits and cost-effectiveness of making such a personal professional available to families with YOPD is now warranted.

### Strengths and limitations

This study is the first exploratory study identifying the impact of PD on current and former partners of a person with YOPD. A qualitative study approach enabled us to collect in-depth information about views and experiences, offering insights from the lived experiences of partners of people with YOPD. In addition, the inclusion of not only current partners of people with YOPD but also of former partners allowed us to retrieve a more detailed picture. By including ex-partners’ views and experiences, we wanted to guarantee that a comprehensive picture of the impact of YOPD is given, including those cases in which a breakdown of the relationship has taken place.

However, our study was not without limitations. First, the use of convenience sampling limits the generalizability of our study findings. As in many other studies [35, 36], despite our efforts, we failed to include participants with a cultural background other than European. Moreover, the majority of participants received a higher level of education. The impact on (ex-)partners of people with YOPD may vary depending on socio-economic background and level of education, with some studies arguing that a lower level of education may be related to a higher level of caregiver burden. However, since our study does not quantify the severity of the caregiver burden, but merely provides an overview of the aspects of life affected by the disease, these differences may be less relevant within the context of our study[37, 38]. Moreover, (ex)partners who volunteered in our study may be different than the general population of (ex-)partners caring for a person with YOPD. It is reasonable that (ex-)partners who feel overburdened or who are in a less stable relationship with the person with YOPD, are less likely to participate in research. Another limitation is that the study does not include a quantitative measurement of the perceived caregiver stress and burden among the included caregivers. It is reasonable that the presence of a higher perceived burden impacts the partner’s experience. In addition, it is evident that disease progression and severity impact the experience of caregivers as certain challenges only occur later, such as feeling housebound and lack of freedom due to increased dependency of the person with PD on the caregiver for performing activities of daily living. Future research should, therefore, explore the role of disease stage and severity on caregivers’ experiences.

### Implications for future research

This research has several implications for future research on the impact of PD on partners of people with YOPD. First, as disease progression is accompanied by greater motor impairments and loss of independency, the impact of PD becomes more noticeable and visible throughout the disease course. Future longitudinal research is, therefore, needed to assess the impact of PD through disease stages to examine how caregiver burden and stress evolve over time. Future research should also explore the needs of partners of people with YOPD for support allowing for the development of age-specific interventions that address the different challenges encountered by people caring for a person with YOPD compared to those caring for a person with PD later in life. Additionally, future research should provide further insights into a variety of variables (e.g. psychological wellness, social context and motor symptoms of the person with YOPD) that could potentially predict caregiving experiences among (ex-)partners of people with YOPD, preferably in a quantitative manner. Within this research, it is important to also further explore differences in caregiving experiences between current partners and ex-partners. Moreover, further qualitative research should aim to gain insight into relevant intercultural differences that may play a role in the perceived impact that YOPD has on the daily lives of (ex)partners. Finally, in this study only (ex-)partners of people with YOPD were included. It remains unclear how children of people with YOPD experience the impact of PD and how certain challenges and struggles they encounter can be reduced or prevented.

### Implications for healthcare professionals

The results of this study have important implications for the clinical practice related to the challenges that partners of people with YOPD face. Being aware of similarities as well as differences between younger and older informal caregivers is key to reduce and prevent caregiver burden. Assessing the impact of PD on various life domains, including work and family life, is recommended. Moreover, healthcare professionals should inquire about the psychosocial and psychological burden that caregivers experience and advise them on counselling suited to their situation. In addition, people with YOPD and their caregivers could potentially benefit from a personal professional, to monitor their at-home situation and provide them with personalized advice on how to overcome their challenges.

### Supplementary Information

Below is the link to the electronic supplementary material.

Supplementary file1 (PDF 160 KB)

## Data Availability

The data generated during the present study are not available due to the inability to make the qualitative data available in a pseudonymized manner.
