# Supplementary material for: Unveiling the invisible: a qualitative interview study on the impact of young onset Parkinson’s disease on (ex-)partners
Source: J Neurol. 2024 Jun 11;271(8):5312–25. doi: 10.1007/s00415-024-12474-2 (PMC11319367; doi:10.1007/s00415-024-12474-2)
Supplement: Supplementary file 1 — Supplementary file1 (PDF 160 KB) [file 415_2024_12474_MOESM1_ESM.pdf]

Unveiling the invisible: a qualitative interview study on the impact of young onset Parkinson's disease on (ex-)partners

Willanka Kapelle[1*], Angelika D. Geerlings[1], Inge Mutsaers[2], Bastiaan R. Bloem[1], Marjan J. Meinders[1], Bart Post[1]

[1] Radboud university medical center; Donders Institute for Brain, Cognition and Behaviour; Department of Neurology; Center of Expertise for Parkinson & Movement Disorders; Nijmegen, The Netherlands
[2] Inge Mutsaers Research & Writing, Nijmegen, The Netherlands

Journal of Neurology

***Corresponding author**

Willanka M. Kapelle, MD

Email: Willanka.Kapelle@radboudumc.nl

INTERVIEW GUIDE

Main question

What is the impact of YOPD on the lives of (ex-)partners of people with YOPD?

Sub-questions

The interview will be divided into three distinct parts:

1. Diagnosis
2. Current life
3. Future

1. Diagnosis

- Trajectory of getting diagnosed
  - What was the trajectory like?
  - How long did it take for you to get a diagnosis?
  - What was your experience regarding your diagnosis?
  - How did the message of your diagnosis come across?
- Shortly after diagnosis
  - What were your biggest concerns shortly after diagnosis?
  - What were your needs after diagnosis?
  - What were your experiences regarding information provision and support, both for yourself and your (ex-)partner?
  - To what extend did you and your (ex-)partner receive (psychological) support after diagnosis?
  - How did your diagnosis affect your expectations for the future
- Role of the (ex-)partner after diagnosis
  - Which steps did you take after your diagnosis?
  - Which role did you take on after diagnosis?
    - Has that role changed over the course of the disease?
    - To what extend do you feel like you are a caregiver to your (ex-)partner? How would you describe this role yourself?

2. Current life

- Private life
  - Impact on the (quality of the) relationship
    - In which ways does YOPD impact your relationship?
    - What is the interaction/communication like between you two?
      - Have there been any changes?
      - What is your mutual understanding like?
    - How have roles and responsibilities changed at home?
    - How has the YOPD impacted independence, autonomy or equality within the relationship?
    - How has YOPD impacted physical contact and sexuality?
  - Impact on (the wellbeing of) children and family dynamics
    - How do your children experience YOPD?

            - How does YOPD impact their lives and well-being?
        - What is the interaction like between you, your (ex-)partner and your children? What has changed?
        - What has changed in the role of you and/or your partner in raising your children?
- Impact of physical wellbeing of the person with YOPD
    - In which ways do you provide aid or support?
    - How do your (ex-)partner's physical complaints impact your life?
    - Which YOPD symptoms are most difficult for you to deal with, or have the most impact on you?
    - How do you perceive the effect of the medication on your (ex-) partner?
        - Are there any side effects that impact you a lot?
    - In case of DBS surgery
        - What were your experiences regarding the surgery?
        - How did your life change after the surgery?
        - How do perceive quality of life before and after surgery? Are you facing different challenges now?
    - In case of pump placement
        - How did your life change after pump placement?
        - How do perceive quality of life before and after pump placement? Are you facing different challenges now?
- Impact of psychological or cognitive changes in the person with YOPD
    - Have you noticed any psychological or cognitive changes in your (ex-) partner? If yes:
        - What do you notice?
        - What do these changes look like? Can you provide an example?
        - How do these changes impact you?
            - How do you cope with them?
    - What is the most challenging aspect to you? What do you find most difficult?
- Own physical and psychological well-being
    - Have you experienced any psychological or physical complaints that could be caused by your partner having YOPD?
    - How would you describe your own coping strategy?
    - To what extend have you been able to accept your situation?
    - How has YOPD changed you as an (ex-)partner or als a person?
- Social life
    - Has YOPD impacted your social life? If yes:
        - How and why?
        - How do you cope with that?
        - To which extend do the people around you impact the way that you experience the impact of YOPD on your life? How?
        - Which knowledge should the people around you have about YOPD?
- Working life
    - To what extend does YOPD impact your working life or how you approach your working life?
    - Which adjustments have you had to make to your working life?

    - What were your experiences regarding said adjustments?
    - What are your experiences regarding the employment trajectory of your (ex-)partner?
- Finances and living
    - To what extent has YOPD impacted your finances?
    - Are your finances a source of worry?
    - To which extend has YOPD impacted your living situation?
- Care and peer-to-peer support
    - What are your experiences regarding the care that your (ex-)partner has received? What could be improved?
    - What are your experiences regarding the support that you have received? What did this support entail?
    - What are your needs regarding support? What could be improved?
    - Have you arranged individual support for yourself?
        - In what way has that helped you?
    - Do you have any contact with peers?
        - What are the perceived benefits?
        - Why do you or do you not feel the need for peer-to-peer support?
    - Has caring for your partner been beneficial to you in any way?
- General questions
    - Which aspect of having an (ex-)partner with YOPD has the biggest impact on your life?
    - Which aspect of your life is most affected by YOPD?
    - What do you find most challenging?

3. Future

- When you think about the future, what comes to mind?
- How does YOPD impact your (expectations about) the future?
- What are you most worried about?
